# Sperm Quality and Welfare of Sexually Mature Boars Supplemented with Partially Fermentable Insoluble Fiber

**DOI:** 10.3390/life15101597

**Published:** 2025-10-13

**Authors:** Daniela Ferreira de Brito Mandu, Vivian Schwaab Sobral, Juliana Cristina Rego Ribas, Maria Fernanda de Castro Burbarelli, Cristiny Santos Braga, Rodrigo Garófallo Garcia, Ibiara Correia de Lima Almeida Paz, Claudia Marie Komiyama, Fabiana Ribeiro Caldara

**Affiliations:** 1 School of Agricultural Science (FCA), Federal University of Grande Dourados (UFGD), Dourados 79824-900, MS, Brazil; daniela.mandu@outlook.com (D.F.d.B.M.); mariaburbarelli@ufgd.edu.br (M.F.d.C.B.); cris_tiny98@yahoo.com.br (C.S.B.); rodrigogarcia@ufgd.edu.br (R.G.G.); claudiakomiyama@ufgd.edu.br (C.M.K.); 2 Agroceres PIC, Rio Claro 13502-741, SP, Brazil; vivian.sobral@agroceres.com (V.S.S.); juliana.ribas@agroceres.com (J.C.R.R.); 3 Faculty of Veterinary Medicine and Animal Sciences (FMVZ), São Paulo State University (UNESP), Botucatu 18618-970, SP, Brazil; ibiara.paz@unesp.br

**Keywords:** behavior, feed restriction, male, satiety, semen, short-chain fatty acid

## Abstract

Dietary fiber plays an important role in animal nutrition by influencing gut health, feed intake, and metabolism. In swine production, studies suggest that fibers may also affect reproductive traits, but findings remain inconsistent, especially in adult boars. This study evaluated the effects of partially fermentable insoluble fiber (PFIF) on semen quality, behavior, and general health of adult boars. Thirty animals were assigned to a completely randomized design with two treatments: (1) CON: no fiber supplementation, and (2) PFIF: fiber supplementation (35 g/animal/day). Fiber was provided once daily for 120 consecutive days. During the period, semen was collected weekly and analyzed macroscopically and microscopically using the Computer-Assisted Sperm Analysis (CASA) system. Behavior was recorded weekly, one and three hours after feeding, based on a pre-established ethogram. Feed intake, perineal, and fecal scores were also evaluated. Fiber supplementation did not affect total motility, progressive motility, sperm concentration, fecal or perineal scores, or behavior. However, improvements were observed in sperm kinematics, with higher straight-line distance (DSL), linearity (LIN), and straightness (STR), as well as a tendency for increased straight-line velocity (VSL) and wobble (WOB). Conversely, a higher incidence of proximal cytoplasmic droplets was recorded in the fiber group, indicating more sperm maturation defects. Supplemented animals also showed reduced feed intake compared with controls, suggesting a satiety effect of the fiber. In conclusion, PFIF supplementation (35 g/animal/day offered once daily) in adult boars produced mixed outcomes, with improved sperm kinematics but increased maturation defects and only minor changes in feeding behavior, indicating a limited and inconsistent physiological response.

## 1. Introduction

Advances in reproductive technologies and genetic improvement have enabled the selection of boars with high spermatogenic capacity, contributing significantly to the overall productivity of the swine industry [1]. Artificial insemination, widely adopted in modern pig production, achieves fertilization rates exceeding 93% in sows, which highlights the crucial role of boars in maintaining reproductive efficiency at the herd level. Nevertheless, factors related to nutritional management, behavior, and animal welfare, which are closely associated with semen quality [2], still require more in-depth investigation.

Although diets for adult boars are formulated to meet their requirements for energy, amino acids, vitamins, and minerals [3], the routine practice of feed restriction, applied to control body condition, may compromise satiety. This limitation can promote stress and stereotypic behaviors, negatively affecting welfare and potentially impairing reproductive function [4]. In this context, dietary fiber inclusion emerges as a promising nutritional strategy, with benefits that extend beyond satiety, including modulation of the intestinal microbiota, maintenance of mucosal integrity, immune homeostasis, and potentially improved semen quality [5].

Partially fermentable insoluble fiber is distinguished by its combination of mechanical and fermentable fractions, thereby promoting multiple physiological effects. The non-fermentable fraction regulates digesta transit time and improves fecal consistency, whereas the fermentable fraction is metabolized by large intestinal bacteria, yielding short-chain fatty acids (SCFA) such as acetate, propionate, and butyrate, as well as lactic acid [6,7]. These metabolites contribute to the maintenance of eubiosis, epithelial integrity, nutrient absorption, and modulation of local inflammatory responses.

In addition, the selective fermentation of fiber components by beneficial microorganisms such as *Lactobacillus* spp. enhances lactic acid production, conferring antimicrobial activity against enteric pathogens. This interplay between fiber structure, microbiota, and fermentative metabolism supports the functional stability of the intestinal environment [8]. Increasing evidence also suggests that intestinal microbiota composition influences sperm parameters, including volume, concentration, motility, and morphology, as demonstrated in studies with other animal models [9,10,11].

Despite the growing interest in this topic, research on the effects of eubiotic fiber supplementation in sexually mature boars remains scarce, particularly under commercial conditions where animals are expected to maintain peak reproductive performance for semen dose production. Given the strategic role of boars in herd productivity, even minor improvements in semen quality may result in substantial increases in insemination doses per ejaculate.

Therefore, the present study aimed to evaluate the effects of dietary inclusion of partially fermentable insoluble fiber in adult boars on semen quality parameters, behavior, and general health indicators, with the goal of elucidating the potential of this nutritional strategy to promote welfare and reproductive performance.

## 2. Materials and Methods

### 2.1. Location and Experimental Facilities

The 120-day experiment was conducted at a commercial artificial insemination unit located in the state of Minas Gerais, Brazil, covering the winter and spring seasons. Animals were housed individually in stalls measuring 2.17 m × 0.67 m, equipped with partially slatted floors, individual stainless-steel feeders, and nipple drinkers. The barn was fitted with an automated negative-pressure cooling system with evaporative pads to maintain optimal environmental conditions for boars. Artificial lights were turned off daily at 21:00 h and switched on again at 06:00 h, providing 9 consecutive hours of darkness.

### 2.2. Animals, Experimental Design, and Treatments

Thirty boars from three genetic lines (Synthetic Pietrain (L1), Landrace (L2) and Large White (L3)), with a mean age of 456 ± 228 days and intended for commercial semen dose production, were allocated to a completely randomized design with two treatments, ensuring equal representation of each genetic line in both groups:

(1) Control (CON)—no fiber supplementation;
(2) Partially fermentable insoluble fiber (PFIF)—daily supplementation of 35 g/animal/day of partially fermentable insoluble fiber in the diet.

All boars included in the study underwent veterinary inspection prior to the experiment, and only clinically healthy animals, free from lesions, infectious diseases, or previous semen abnormalities, were enrolled. The boars were part of a routine herd health program, receiving standard vaccinations against reproductive and respiratory pathogens, as well as regular antiparasitic treatments according to farm protocols. Their health status was monitored daily by farm staff and the attending veterinarian throughout the experimental period, and any boar showing clinical signs of illness or injury would have been excluded from the trial.

### 2.3. Feeding Management and Fiber Supplementation

Animals were fed once daily, between 07:00 and 08:00 h, receiving 2.5 ± 0.5 kg of diet per animal, adjusted according to body condition score, and delivered via automated drop feeders. The diet, provided in pelleted form, was specifically formulated for breeding boars and contained 3000 kcal ME/kg, 15% crude protein, 3% ether extract, and 8% crude fiber, with the following minimum levels of essential amino acids: 0.80% lysine, 0.25% methionine, 0.60% threonine, and 0.19% tryptophan, in accordance with the recommendations of Rostagno et al. [12]. The formulation included soybean hulls, soybean meal, wheat bran, wheat gluten, ground corn, degummed soybean oil, sucrose, limestone, kaolin, sodium chloride, dicalcium phosphate, vitamin and mineral premix (including DL-methionine, L-lysine, L-threonine, L-tryptophan, biotin, niacin, folic acid, vitamins A, D, E, C, and B-complex), acidifiers, probiotics, enzymes, antioxidants (BHA, BHT, etoxyquin), yeast cell wall, selenium and chromium chelates, and other additives commonly used in boar diets.

Concomitantly with the provision of the basal diet, animals in the treatment group received a daily supplementation of 35 g of wood-derived vegetable fiber, administered individually together with the regular feed. The fiber used was classified as insoluble and partially fermentable, consisting predominantly of lignocellulose obtained from fresh wood, free of contaminants and mycotoxins, and presenting the following average composition: 59% crude fiber, 78% neutral detergent fiber (NDF), 64% acid detergent fiber (ADF), 25–30% lignin, ~0% energy, 0.9% crude protein, 8% moisture, 1% crude ash, and 1.3% minerals and trace elements. The raw material underwent thermal treatment and fine milling, resulting in standardized particles with sizes ranging approximately from 50 to 120 µm, which provide a large surface area for microbial fermentation in the hindgut. This processing ensures product safety and consistency, while the exact tree species used and additional processing details are proprietary to the manufacturer.

### 2.4. Semen Collection and Quantitative and Qualitative Analyses

Semen collection procedures were initiated immediately after feeding. Two to three times per week, boars were removed from their stalls and individually guided using handling boards, rattles, auditory stimuli (whistles), and, when necessary, hand gestures to encourage movement. The animals were then directed to the holding and collection pens, where a trained operator performed preputial cleaning with paper towels, followed by transfer to the collection area equipped with a dummy and an automated penis fixation system. The pre-sperm fraction of the ejaculate was discarded, and the sperm-rich fractions were collected in sealed insulated containers and transferred to the laboratory via a closed pneumatic tube system for macroscopic and microscopic quality assessment. Based on ejaculate volume, sperm concentration, and standardized sperm content per dose (2 × 10^9^ sperm/dose), the number of possible doses per ejaculate was calculated. Sperm analyses were conducted using a Computer-Assisted Sperm Analysis (CASA) system. Upon completion of collection, each boar was returned to its stall by the same handler, using the same management procedures.

### 2.5. Feed Intake Scoring

Approximately 40 minutes after feed delivery, visual assessments of feed intake were conducted and categorized into three scores: 1 (0–30% of the offered amount), 2 (31–60%), and 3 (>61%). Evaluations were performed by a single trained observer, previously calibrated and blinded to the experimental treatments. The visual scoring of feed intake, based on feeder coverage percentage, was adapted from commercial monitoring practices [13] and adjusted to the categories 1 (0–30%), 2 (31–60%), and 3 (>60%).

### 2.6. Perineal and Fecal Scoring

Visual evaluation of the perineal region was performed weekly using a scale adapted from Kiefer et al. [14], originally developed for sows to monitor the risk of uterine prolapse. Scores ranged from 0 (no swelling), 1 (mild swelling), 2 (moderate swelling), to 3 (severe swelling), with criteria based on visible protrusion and edema.

At the same time, fecal consistency was assessed using visual scores according to the following criteria: 0—normal feces; 1—semisolid consistency; 2—creamy consistency; and 3—watery consistency. An additional 0.5 points was assigned in cases with detectable presence of mucus and/or blood [15]. Coprostasis was assessed simultaneously through visual inspection, and animals showing persistent coprostasis (hard, dry feces with difficult elimination) were registered according to the same scoring system; however, no cases were observed during the experimental period.

### 2.7. Behavioral Assessment

Focal behavioral observation was conducted on site at two distinct time points: one hour and three hours after feed delivery, always performed by the same trained observer. The observational protocol was based on an ethogram adapted from Bernardino et al. [16] (Table 1). During each session, three consecutive samples were collected at two-minute intervals. Recorded behaviors are expressed as the mean of the three observations.

### 2.8. Microclimatic Conditions

Prior to each behavioral assessment, temperature and relative humidity readings were taken from the climate control system panel. In addition, the barn microclimate was periodically monitored and recorded using a temperature and humidity sensor (data logger HT-810, Instrutemp, São Paulo, Brazil) positioned at the center of the facility, near the climate control system sensor. Throughout the experimental period, microclimatic conditions inside the barn showed some variation, with temperatures ranging from 18 to 25 °C and relative humidity from 40 to 70%.

### 2.9. Statistical Analysis

Semen evaluation data were tested for normality of residuals and homogeneity of variances, using the Shapiro–Wilk test and Levene’s test, respectively. When assumptions were not met, data were appropriately transformed prior to analysis to ensure the validity of the statistical models. Original or transformed data were then subjected to analysis of variance using the MIXED procedure of SAS (version 9.4, SAS Institute Inc., Cary, NC, USA) [17]. The F-test was performed, and when significant, since only two groups were tested, it was assumed that they differed from each other. Animal age, order of collection, and the number of days between collections were included in the statistical model as covariates when their effects were significant.

Feed intake, perineal, and fecal scores were considered categorical variables (frequencies) and analyzed using non-parametric statistics by means of Chi-square test of independence or Fisher’s exact test with the FREQ procedure of SAS (version 9.4, 2015). Fisher’s test were adopted when frequencies were lower than 5%. Data in the tables are presented as percentages of responses obtained in each evaluation for the treatments.

Behavioral data were analyzed using the GLIMMIX procedure of SAS (version 9.4, SAS Institute Inc., Cary, NC, USA). As these data did not meet the assumption of residual normality, they were transformed using the LOGNORMAL matrix. In this approach, GLIMMIX models the logarithm of the response variable as a normally distributed random variable, with mean and variance estimated on the log scale, thereby assuming a normal distribution. Animal age was included in the model as a covariate. Least square means were compared using the pdiff ilink option of the GLIMMIX procedure, which provides estimates adjusted by the inverse link function. Data are expressed as percentages of the frequency of each behavior in relation to all behaviors observed during the evaluation period. For all statistical analyses conducted in this study, a significance level of 5% was adopted.

## 3. Results

### 3.1. Quantitative and Qualitative Semen Parameters

Supplementation with partially fermentable insoluble eubiotic fiber did not significantly affect conventional semen parameters, including total motility, progressive motility, and sperm concentration (*p* > 0.05). However, an effect was observed on sperm kinematic variables assessed by the CASA system. Boars of the PFIF group exhibited greater straight-line distance (DSL; *p* = 0.0066), higher linearity (LIN; *p* = 0.0010), and greater straightness (STR; *p* < 0.0001), as well as a trend toward increased straight-line velocity (VSL; *p* = 0.063) and wobble (WOB; *p* = 0.0949), compared with the CON group. Conversely, a lower percentage of morphologically normal sperm with respect to proximal droplets (GOTAP; *p* = 0.0029) was observed, indicating a higher frequency of this defect in supplemented animals (Table 2).

### 3.2. Feed Intake, Fecal, and Perineal Scores

With respect to feed intake score, although most animals in both groups presented score 3 (consumption greater than 61% of the feed offered), a significantly higher frequency of lower scores (1 and 2) was observed in the fiber-supplemented group (Table 3). No significant differences (*p* > 0.05) were observed between treatments for fecal and perineal scores. Regarding fecal score, most animals presented score 0, indicating normal fecal consistency in both groups. Scores 2 and 3, which reflect altered fecal consistency, were recorded only in the CON group and at very low frequency (0.08% each), and were absent in the PFIF group. For perineal score, most animals presented score 0 (no swelling), with lower frequencies of scores 1 and 2 (mild to moderate swelling). Score 3 (severe swelling) was not recorded in either group (Table 3).

### 3.3. Behavior

Behavioral analysis of the boars revealed no significant differences between treatments for any of the evaluated categories (*p* > 0.05). In both groups, the most frequently observed behaviors were resting (lying down) and standing still, followed by stereotypic behaviors. The latter were numerically more prevalent in the CON group (23.67%) compared with the PFIF group (17.92%), although the difference was not statistically significant (*p* = 0.225). Social behaviors, both positive and negative, as well as sexual behavior, were recorded at low frequencies (< 0.5%) in both treatments, which is consistent with the individual housing in stalls. The category “other behaviors” remained stable between groups (Table 4).

## 4. Discussion

The hypothesis of this study was based on evidence that both fermentable and non-fermentable fractions of dietary fiber can promote satiety and reduce aggressive and stereotypic behaviors [3,18,19], improve intestinal health through the production of short-chain fatty acids (SCFA) [8,20], modulate inflammation, and preserve epithelial integrity [21], mechanisms that collectively support systemic homeostasis and may influence spermatogenesis [11,22,23,24,25]. However, the results obtained were limited and did not fully confirm this hypothesis.

Most studies reporting beneficial effects of fiber on male reproductive function have been conducted in young or growing animals, a stage in which spermatogenesis is more sensitive to nutritional and metabolic variations [22,23]. In sexually mature boars with already normal semen parameters, as in the present study, the physiological margin of response tends to be narrower. This was confirmed by the values of semen volume, motility, and morphology, which were consistent with boars in full reproductive performance [26,27].

The type of fiber tested is another critical factor. The lignocellulose used, structurally similar to wheat bran—composed mainly of arabinoxylans and cellulose with 85–90% insoluble fraction—has low fermentability [8,28]. While finely ground wheat bran can increase SCFA production and exert beneficial effects on the microbiota and intestinal transit [29,30], lignocellulose, even when ground, remains less accessible to microbial fermentation [21,31]. Without robust SCFA production, expected systemic effects such as inflammatory modulation and metabolic support are attenuated [11,32]. Furthermore, the tested dose (35 g/day, equivalent to 1.4–1.75% of feed intake and 0.009–0.012% of body weight) was relatively low compared with other models. In laying hens (approximately 2 kg body weight), dietary inclusions of 2–4% correspond to 0.12–0.20% of body weight and have produced marked changes in microbiota composition and SCFA levels [33]. It is therefore plausible that the low fermentability and reduced dosage, combined with the physiological particularities of adult boars, limited metabolite production to levels insufficient for prominent systemic effects.

Nevertheless, some findings are noteworthy. Despite the higher frequency of proximal droplets, indicative of epididymal maturation failure [34,35,36], improvements were observed in sperm kinematic parameters such as LIN, STR, and DSL. These indices reflect more progressive and efficient movement, associated with higher fertilizing potential [37,38,39]. It is possible that even partial fermentation of insoluble fiber provided additional energetic substrates (via acetate and propionate), sufficient to optimize sperm metabolism and favor linear movement. Conversely, the acceleration of intestinal transit induced by insoluble fiber may have reduced the absorption of key micronutrients essential for epididymal sperm maturation, such as zinc and selenium—critical for chromatin stabilization, flagellar integrity [40,41,42,43], and antioxidant defense—along with copper, manganese, and antioxidant vitamins (E, C, and A) [44,45,46]. At the same time, the lower availability of butyrate may have limited anti-inflammatory modulation and epithelial support, further compromising the epididymal environment required for proper cytoplasmic droplet migration and final sperm maturation [9,25,47]. These combined factors help explain the dissociation observed: improved sperm kinematics, but impaired epididymal maturation indices. In summary, supplementation with partially fermentable insoluble fiber proved effective in enhancing sperm motility parameters, but was insufficient to mitigate morphological alterations related to proximal droplets, suggesting a partial physiological response or the need to adjust fiber dosage, duration, or dietary combination.

From a behavioral perspective, only modest effects were observed. Supplementation slightly reduced immediate feed intake, in line with the literature associating insoluble fibers with satiety through gastric distension and the release of GLP-1 and PYY [7,8,18], but without noticeable impact on overall behavior. The absence of changes in fecal or perineal scores may be related to the physical–structural properties of the fiber. Finely ground particles lose water-holding capacity (WHC) due to structural collapse [48], thereby reducing their mechanical and laxative effects in the gastrointestinal tract. This would explain the stability of fecal consistency and, consequently, perineal scores, since fecal moisture influences intra-abdominal pressure and the integrity of this region. Regarding welfare, the frequency of stereotypies was only numerically lower in supplemented animals (17.9% vs. 23.7%), suggesting a marginal effect, likely constrained by intensive housing, which imposes social and locomotor restrictions and masks potential satiety-related benefits. The high incidence of stereotypic behaviors underscores the need for integrated approaches to improve welfare, in line with current regulations that require environmental enrichment and the replacement of stalls with group housing [49,50].

Thus, the absence of significant responses should be interpreted as the outcome of a combination of factors, including the physiological stage of the animals, the low fermentability and dosage of the fiber used, and the housing conditions. Nevertheless, the findings highlight mixed effects: improvements in kinematics, an increase in morphological defects, and a modest impact on feeding behavior.

An additional aspect to consider is the potential influence of ambient temperature. Although environmental effects were not the main objective of this study, all animals were kept in climate-controlled facilities that limited major fluctuations. During the experimental period, maximum values occasionally reached 25 °C, slightly above the traditionally recommended comfort zone for adult boars (around 15–20 °C) [51]. However, recent evidence by Raber et al. [52] demonstrated that sexually mature boars of different breeds spent most of their inactive time at approximately 25.5 °C and preferred lying positions at about 25.9 °C, suggesting that their effective thermal comfort zone may extend to higher temperatures than previously assumed. In this context, the modest variation in barn microclimate observed in our trial is unlikely to have negatively influenced the animals. This interpretation is further supported by the absence of changes in behavior, fecal consistency, and seminal parameters, including sperm morphology (abnormalities), with only discrete improvements detected in sperm kinematics. Therefore, the lack of more pronounced effects of insoluble fiber supplementation should be attributed primarily to its limited fermentability, the relatively low inclusion level, and the consequent low short-chain fatty acid production, rather than to thermal environmental interference.

Considering the effects observed in this study, future investigations should include the assessment of sperm membrane and DNA integrity, as well as acrosomal status and oxidative stress markers, to determine potential protective effects against cellular damage. Although improvements in sperm trajectories and kinematic indices are promising, they do not guarantee enhanced fertility if other limiting factors such as morphology or DNA integrity are compromised. In addition, statistical significance does not necessarily reflect practical relevance; therefore, effect sizes should be carefully examined to determine whether such differences are biologically meaningful under field conditions. The measurement of reproductive hormones, such as testosterone, could further clarify possible metabolic–hormonal interactions, while in vivo fertility trials remain essential to verify whether the observed improvements in kinematic parameters translate into higher conception rates. Finally, characterization of the intestinal microbiota and quantification of SCFA in adult boars would help establish a more direct mechanistic link between fiber supplementation, intestinal metabolism, and semen quality.

### Limitations of the Study

This study presents some limitations that should be considered when interpreting the results. The trial was conducted in sexually mature boars with already optimal semen parameters, which may have reduced the margin of response. In addition, the type and low dose of fiber used (lignocellulose, 35 g/day) limited fermentability and short-chain fatty acid production, attenuating potential systemic effects. Intensive housing conditions may also have masked behavioral outcomes. Finally, although improvements in sperm kinematics were observed, further studies are needed to assess sperm integrity and fertility under field conditions.

## 5. Conclusions

Supplementation with partially fermentable insoluble fiber in adult boars resulted in mixed effects on semen quality and feeding behavior. Improvements were observed in kinematic parameters; however, this was accompanied by an increased frequency of proximal cytoplasmic droplets. In addition, a slight reduction in immediate feed intake was detected, consistent with enhanced satiety, but without significant changes in fecal or perineal scores or in other behavioral parameters evaluated. These findings indicate that, in adult males, partially fermentable insoluble fiber exerts a partial and non-uniform physiological influence, highlighting the need for further studies assessing different types and inclusion levels of fiber, as well as additional parameters.

## Figures and Tables

**Table 1:** Ethogram used for the behavioral analysis of boars supplemented or not with partially fermentable insoluble fiber in the diet.

Behavior	Description
Negative social interaction	Animal displaying aggressive behavior such as biting, mounting, headbutting, or performing any social behavior that disturbs or bothers another animal.
Positive social interaction	Animal sniffing, nuzzling, sucking, or gently touching another animal without triggering aggressive responses from the other individual.
Sexual behavior	Chewing without the presence of food, excessive salivation, with or without the Flehmen reflex (head elevation and curling of the upper lip). Mounting simulation directed at pen structures or exhibiting dorsal arching, with repetitive copulatory movements, with or without ejaculation.
Stereotyped behavior	Animal performing repetitive activities with no apparent function (e.g., licking the floor and/or the feeder, biting the bars of the crate, chewing air, activating the drinker without water intake, head shaking).
Sitting	Animal in a sitting position (hindquarters and forepaws on the floor).
Lying	Animal in lateral or sternal recumbency, not engaging in any activity, with eyes open or closed.
Standing still	Animal standing still, with the soles of 3 or 4 limbs on the floor.
Other active behavior	Animal drinking water, eating feed, exploring the environment with the snout, moving within the pen, urinating, or defecating.

**Table 2:** Macroscopic and microscopic ejaculate parameters of boars supplemented or not with partially fermentable insoluble fiber in the diet.

Semen Characteristics	CON	PFIF	SEM	*p*-Value
Volume (mL)	209.63	211.99	2.877	0.677
Sperm concentration (× 10^6^/mL)	440.00	430.00	0.006	0.142
Total sperm count (×10^9^)	88.65	87.20	1.263	0.562
Total motility (%)	95.09	95.40	0.135	0.256
Progressive motility (%)	86.62	87.36	0.285	0.196
DCL—Curvilinear displacement (µm)	111.28	110.77	0.908	0.780
DAP—Average path displacement (µm)	55.92	56.58	0.428	0.380
DSL—Straight-line displacement (µm)	40.77	42.83	0.377	0.006
VCL—Curvilinear velocity (µm/s)	178.76	176.14	1.560	0.406
VAP—Average path velocity (µm/s)	90.12	90.42	0.733	0.836
VSL—Straight-line velocity (µm/s)	66.72	69.04	0.619	0.063
STR—Straightness	73.32	75.83	0.324	0.000
WOB—Wobble	51.94	52.54	0.177	0.095
LIN—Linearity	38.95	40.57	0.246	0.001
Total sperm defects (%)	13.37	14.28	0.295	0.127
Proximal Droplet (%)	4.41	3.71	0.116	0.003
Distal Droplet (%)	5.53	5.87	0.132	0.203
Possible doses (n)	35.90	34.69	0.499	0.228

CON—control group; PFIF—partially fermentable insoluble fiber; SEM—Standard Error of the Mean.

**Table 3:** Feed intake, fecal, and perineal scores of boars supplemented or not with partially fermentable insoluble fiber in the diet.

Parameter	Score	CON	PFIF	*p*-Value
Feed intake score	1	0.07%	0.59%	0.0002
	2	0.33%	1.45%	
	3	99.60%	97.95%	
Fecal score	0	99.84%	100%	0.1506
	2	0.08%	0.00%	
	3	0.08%	0.00%	
Perineal score	0	76.47%	73.33%	0.4283
	1	23.53%	26.67%	

CON—control group; PFIF—partially fermentable insoluble fiber.

**Table 4:** Behavioral frequency (%) of boars supplemented or not with partially fermentable insoluble fiber in the diet.

Behavior	CON	PFIF	SEM	*p*-Value
Negative social interaction	0.00	0.05	0.000	1.000
Positive social interaction	0.24	0.13	0.286	0.292
Sexual behavior	0.17	0.41	0.281	0.154
Stereotyped behavior	23.66	17.92	0.170	0.225
Sitting	9.06	6.87	0.281	0.116
Lying	32.49	34.68	0.153	0.480
Standing still	27.99	34.28	0.166	0.117
Other active behavior	6.39	5.66	0.114	0.742

CON—control group; PFIF—partially fermentable insoluble fiber; SEM—Standard Error of the Mean.

## Data Availability

Additional research data, including further details on protocols, analytic methods, raw data, and processed data, will be made available upon request to interested researchers.

## Abbreviations

The following abbreviations are used in this manuscript:

Short-chain fatty acid	SCFA
Control treatment	CON
Partially fermentable insoluble fiber	PFIF
Computer-Assisted Sperm Analysis	CASA
Curvilinear displacement	DCL
Average path displacement	DAP
Straight-line displacement	DSL
Curvilinear velocity	VCL
Average path velocity	VAP
Straight-line velocity	VSL
Straightness	STR
Wobble	WOB
Linearity	LIN
